# Doctors and the War

**Published:** 1915-10-02

**Authors:** 


### The Hospital

The Workers' Newspaper of Administrative Medicine and Institutional Life, Administration, National Insurance and Health.

No. 1529, Vol. LIX. SATURDAY, OCTOBER 2, 1915. PRICE ONE PENNY.

230055

### DOCTORS AND THE WAR.

The position in relation to the supply of doctors for the armies now on active service or preparing for this duty is gradually becoming more precise, and though widespread and considerable sacrifices will be necessary, the profession, we are sure, will prove equal to the occasion. If the claim is great, so also is the honour. And though the more conspicuous sphere is necessarily reserved for the younger members, all must contribute something if the tribute of professional co-operation and helpfulness is to be complete.

A very definite step in advance is the authoritative announcement of the numbers required. We now learn that the Director-General needs, before the end of the present year, at least 2,500 more medical men ready to take commissions and to go wherever they may be needed. This means at the very least one-third of the members of the profession who are within the military age, and it is on these that the immediate summons falls. To say that the position is exceptional is a mere commonplace. Apart from other great and worthy ends, the nation is fighting for its very existence, and has as its chief enemy the most formidable military organisation that the world has ever seen. The issue is tremendous, and will govern the course of civilisation in Europe for many a day. That this country may play in the great struggle for freedom a part equal to her history and her duty she must raise armies far outnumbering any figures deemed possible prior to the war. Without doctors these armies as fighting organisations are impossible. The profession therefore stands in a very special position, and the degree of opportunity is the measure of the extent of the obligation. The service now asked will write history in no doubtful terms, and will enrich both our patriotic and our professional traditions. The request is made to free men, and the answer will be an index of the interpretation these will place on the statement of their country's need.

Now the necessity is stated in plain words and figures it is possible to take the steps necessary to meet it. There has been no want of promptness in this direction. Already the country is divided into districts in each of which has been constituted a War Emergency Committee. From the Central Committee the local organisations have been informed of the numbers which each district is estimated to produce, and the answer to this intimation must be secured by the practitioners in the district concerned. Local meetings of the profession have been summoned, and some of these have been addressed by representatives of the War Office or by prominent members of the profession. In short, active measures are in progress to secure that there is brought prominently before every practitioner the urgency and acuteness of the present emergency. It is too soon to state exactly what degree of success has attended these operations, but we can certainly say that the results are encouraging, and it is impossible to doubt that now the exact requirements of the Army are known the profession will see that these are fully satisfied.

A helpful step towards the desired end is the decision of the Central Emergency Committee to draw the attention of the governing bodies of hospitals to the undesirability of giving resident appointments to young men fit for military service. This action has been repeatedly urged in our columns, and we hope to see a general response to it. Without question, the younger men in practice have felt the call upon them for sacrifice to be unfair so long as newly qualified juniors were invited to accept resident positions of relatively long duration. The way to remove from this grievance its *prima facie* warrant is to announce either that such positions are not open to men of military age, or that they will be given to those ready to accept commissions, and for brief periods only. This latter course might well apply to those who have recently passed the final examination and who desire some practical experience before undertaking active military duties. As we have pointed out before, here is an opportunity for the hospitals actively to co-operate with the military authorities, and to associate themselves still further with the nation's task. Already the hospitals have done much in the placing of all their resources at the disposition of the War Office for the care of the sick and wounded soldiers. There is still another opportunity for corporate recognition of the country's

W1 N069X

need, and it is to be found in the direction we have above indicated.

For the doctors who, on account of age or other reasons, remain at home there are also obligations of service. The first of these is perhaps a duty to the colleagues who have left all for the sake of the common cause. This is a consideration to which necessarily much attention is being paid. The aim is to see that when the practitioner returns from the wars he shall find his practice and means of livelihood reasonably intact. Of course such an end in each individual case can only be secured by the loyal co-operation of neighbouring practitioners, and it is no surprise to learn that this co-operation is being widely secured. Quite rightly, too, the arrangements are being organised on a practical business basis, and are not to be left to a sentiment which time and the event might unduly strain. Last of all, it is recognised that in these proposals the public must take some part, and an appeal has been issued for consideration of the strain which must needs fall upon the doctors left to conduct civil practice.

